# Developmental or adult-onset deletion of neurotensin receptor-1 from dopamine neurons differentially reduces body weight

**DOI:** 10.3389/fnins.2022.874316

**Published:** 2022-09-23

**Authors:** Patricia Perez-Bonilla, Jariel Ramirez-Virella, Pooja Menon, Eva Troyano-Rodriguez, Sydney K. Arriaga, Anna Makela, Raluca Bugescu, Michael J. Beckstead, Gina M. Leinninger

**Affiliations:** ^1^Neuroscience Graduate Program, Michigan State University, East Lansing, MI, United States; ^2^Department of Pharmacology and Toxicology, Michigan State University, East Lansing, MI, United States; ^3^Department of Physiology, Michigan State University, East Lansing, MI, United States; ^4^Aging and Metabolism Research Program, Oklahoma Medical Research Foundation, Oklahoma City, OK, United States; ^5^Oklahoma City Veterans Affairs Medical Center, Oklahoma City, OK, United States

**Keywords:** ventral tegmental area (VTA), feeding, locomotor activity, obesity, energy balance

## Abstract

Central neurotensin signaling via neurotensin receptor-1 (NtsR1) modulates various aspects of physiology, including suppressing feeding and promoting locomotor activity that can support weight loss. However, it remains unclear when and where NtsR1 expression contributes to control of body weight vs. other effects. We previously showed that activating ventral tegmental area (VTA) dopamine (DA) neurons that express NtsR1 promotes weight loss. We therefore hypothesized that deleting NtsR1 from DA neurons would promote weight gain by increasing food intake and decreasing physical activity. In contrast, developmental deletion of NtsR1 from DA neurons (by crossing *DAT^Cre^* mice with *NtsR1^flox/flox^* mice) had no impact on the feeding or body weight of mice fed a chow diet, though it augmented locomotor activity. Developmental deletion of NtsR1 from DA neurons protected mice from diet-induced obesity, but not *via* altering feeding, physical activity, or energy expenditure. Given that NtsR1 may exert distinct roles within development vs. adulthood, we then examined the impact of adult-onset deletion of NtsR1 from VTA DA neurons. We injected adult *NtsR1^flox/flox^* mice in the VTA with adeno associated virus to Cre-dependently delete NtsR1 in the VTA (*VTAR1^Null^* mice) and compared them to mice with intact NtsR1 (Controls). Again, in contrast to our hypothesis, *VTAR1^Null^* mice gained less weight than Controls while on normal chow or high fat diets. Moreover, *VTAR1^Null^* mice exhibited blunted feeding after fasting, suggesting a role for NtsR1 in adult VTA DA neurons in coordinating energy need and intake. Altogether, these data suggest that intact expression of NtsR1 in DA neurons is necessary for appropriate regulation of body weight, but a lack of NtsR1 in the developing vs. adult DA system protects from weight gain *via* different mechanisms. These findings emphasize the need for temporal and site-specific resolution to fully understand the role of NtsR1 within the brain.

## Introduction

Energy balance is the coordinated regulation of calories consumed and calories expended. Organisms take in energy by ingesting foods and caloric liquids, and expend energy through physical movement and basal metabolism. Brain control of ingestive and locomotor behaviors, however, have been influenced by environment, namely the availability of highly palatable energy dense foods and increasingly sedentary lifestyles. These factors have led to the rise in overweight and obese individuals worldwide (Haslam and James, 2005). Obesity elevates the risk of developing severe chronic conditions such as cardiovascular disease, type-2 diabetes, kidney disease, cancer and disability, and is responsible for approximately 4 million annual deaths (Afshin et al., 2017; Gregg and Shaw, 2017). Yet, there are few efficacious pharmacotherapies for treating obesity due to the heterogeneity of the disease, and incomplete understanding of how the brain regulates energy balance (Hainer et al., 2008; Mayoral et al., 2020). Elucidating how the brain coordinates feeding, physical activity and energy expenditure is critical to understanding normal physiology and may aid in identifying approaches to support sustained weight loss.

Neuropeptides and their receptors have surfaced as important regulators of body weight and as possible systems to target for weight reduction. The neuropeptide neurotensin (Nts) is particularly promising in this regard because administering Nts centrally suppresses feeding and, in certain sites, also increases locomotor activity (Cador et al., 1986; Elliott and Nemeroff, 1986; Schroeder and Leinninger, 2018; Torruella-Suárez and McElligott, 2020). The central weight-reducing actions of Nts are mediated *via* the Neurotensin Receptor-1 (NtsR1), a G-protein coupled receptor typically coupled to G_*q*_ proteins (Tanaka et al., 1990; Hermans et al., 1992). In general, studies of whole body NtsR1 knockout mice support a role for NtsR1 in regulating energy balance, but different strains have produced divergent conclusions about how it modulates locomotor activity, homeostatic feeding, and body weight [reviewed in Schroeder and Leinninger (2018)]. In addition, the whole body developmental deletion in these models may lead to compensatory changes that mask normal action of the receptor (Pettibone et al., 2002; Remaury et al., 2002; Kim et al., 2008; Liang et al., 2010). Further complicating the use of constitutive NtsR1 null mice is that NtsR1 expression varies with age and brain region. NtsR1 is transiently upregulated during gestation and peaks shortly after birth, but is subsequently downregulated as animals reach maturity (Palacios et al., 1988), which suggests that NtsR1 plays different roles in development and adulthood. Within the adult brain, NtsR1 is expressed within the cingulate cortex, midbrain, subiculum and in the hindbrain (Kessler et al., 1987; Moyse et al., 1987; Najimi et al., 2014), but the roles of NtsR1 in these regions may differ. Indeed, activating site-specific Nts- or NtsR1-expressing neurons can modulate physiology ranging from social interaction, locomotor activity, feeding and drinking (McHenry et al., 2017; Woodworth et al., 2017b; Kurt et al., 2019; Torruella-Suárez et al., 2020; Perez-Bonilla et al., 2021). Given the temporal and site-specific expression of NtsR1, it is imperative to systematically examine its role within discrete neuronal populations and at different stages of life.

We have begun to characterize NtsR1-expressing neurons, including those in the ventral tegmental area (VTA) that comprise a subset of the dopamine (DA) neurons in this brain region (Palacios et al., 1988; Lepee-Lorgeoux et al., 1999; Woodworth et al., 2017a,^2018^; Perez-Bonilla et al., 2021). In this population, which we will henceforth refer to as VTA NtsR1 neurons, Nts is generally excitatory and can produce a direct depolarization through NtsR1 (Jiang et al., 1994; Tschumi and Beckstead, 2019). VTA NtsR1 neurons hold promise as targets to modify body weight, as DA neurons in the VTA are essential modulators of feeding and locomotor activity (Zhou and Palmiter, 1995; Narayanan et al., 2010). Moreover, Nts and NtsR1 agonists delivered into the VTA of adult animals restrain feeding and promote DA-dependent locomotor activity (Cador et al., 1986; Hawkins, 1986; Woodworth et al., 2017b). Similarly, we recently showed that activating VTA NtsR1 neurons in normal weight and obese adult mice promotes weight loss by suppressing food intake, increasing locomotor activity and increasing energy expenditure (Perez-Bonilla et al., 2021). We therefore hypothesized that deleting NtsR1 specifically from VTA DA neurons would promote weight gain by increasing food intake while decreasing physical activity and energy expenditure. To address this hypothesis, we developed *NtsR1^flox/flox^* mice and 1) crossed them with *DAT^Cre^* mice to developmentally delete NtsR1 from all DA expressing neurons (*DATR1^Null^*) or 2) injected them in the VTA with an adeno associated Cre (AAV-Cre-GFP) virus to generate adult-onset, site-specific deletion of NtsR1 (*VTAR1^Null^*). Rather than promoting weight gain as we hypothesized, our data show that developmental or adult deletion of NtsR1 from DA neurons protects mice from weight gain, but only deletion from adult VTA DA neurons impacts feeding. These findings emphasize the need for temporal and site-specific examination of NtsR1 expression to understand its contributions to physiology.

## Research design and methods

### Mice

*NtsR1^flox^* mice were generated by the Michigan State University Transgenic and Genome Editing Facility (TEGF). A CRISPR/Cas9 strategy was used to insert loxP sites flanking exon 1 of the genomic *Ntsr1* locus. Correct insertion of both loxP sites in the founder mouse was validated by DNA sequencing. The founder was crossed with C57Bl/6J mice (Jackson Stock #033365) for 3 generations to stabilize the insertion. Subsequent *NtsR1^flox/+^* progeny were intercrossed to generate *NtsR1^flox/flox^* mice for studies. All mice were bred and housed under a 12 h light/12 h dark cycle and were cared for by Campus Animal Resources (CAR). We studied an approximately equal number of male and female mice on the C57Bl/6J background. Cohorts were staggered to control for any potential seasonal effects. Since we did not observe any metabolic or behavioral differences between males and females, both sexes were pooled. All animal protocols were approved by the Institutional Animal Care and Use Committee (IACUC) at Michigan State University and the Oklahoma Medical Research Foundation, in accordance with the Association for Assessment and Accreditation of Laboratory Animal Care and National Institutes of Health.

*Developmental Model*: *NtsR1^flox^* mice were intercrossed with *DAT^Cre^* mice (Jackson Stock #006660) to generate *DATR1^Null^* mice (*DAT*^*Cre*/+^;*NtsR1^flox/flox^)*, which undergo early developmental Cre-mediated deletion of NtsR1 from all DAT-expressing neurons. We also studied (*DAT^+/+^;NtsR1*^flox/flox^*)* littermates as controls, referred to as *NtsR1*^flox/flox^**, that retain NtsR1 expression in all cells. For brain slice electrophysiology experiments, mice were group housed in ventilated standard cages with *ad libitum* access to food and water. Rooms were maintained on a normal 12-h light/dark cycle (lights on at 6:00 AM) with the temperature held at 26°C.

*Adult Model*: Heterozygous *NtsR1*^*flox/*+^ mice were mated to produce homozygous *NtsR1^flox/flox^* mice and *NtsR1*^+/+^ (referred to as wild type, *Wt*) littermates. *NtsR1^flox/flox^* and *Wt* mice were then injected at ∼8 wks of age in the VTA (bilaterally) with either AAV-GFP or AAV-Cre-GFP to generate *NtsR1^flox/flox^* (*VTAR1^Null^*), *Wt^Cre^*, *NtsR1^flox/flox;GFP^* (*VTAR1^GFP^*), and *Wt^GFP^* mice. *VTAR1^Null^* mice thus have an adult-onset deletion of NtsR1 specifically from VTA neurons.

### Gene expression

Male and female 12–16 wk old *NtsR1^flox/flox^* and *Wt* littermates were deeply anesthetized with sodium pentobarbital. Tissue from the VTA was microdissected, snap frozen on dry ice and stored at −80°C. RNA was extracted using Trizol (Invitrogen) and 200 ng samples were converted to cDNA using the Superscript First Strand Synthesis System for RT-PCR (Invitrogen). Sample cDNAs were analyzed in triplicate *via* quantitative RT-PCR for gene expression using Applied Biosytems TaqMan probes for Ntsr1 (Mm00444459_m1), tyrosine hydroxylase (TH; Mm00447557_m1) and GAPDH (Mm99999915_g1) using a BioRad CFX Connect Real-Time System. With GAPDH expression as an internal control, relative mRNA expression values were calculated by the 2^–ΔΔ^Ct method.

### Brain slice electrophysiology

Recordings were done on dopaminergic neurons from 14 to 20-month-old male and female *NtsR1^flox/flox^* (as controls) and *DATR1^Null^* mice. A total of 9 *NtsR1^flox/flox^* cells (4 from males and 5 from females) and 6 *DATR1^Null^* cells (4 from females and 2 from males) were included in the data analysis. On the day of the experiment, mice were anesthetized with 2% 2,2,2-tribromoethanol and transcardially perfused with ice-cold carboxygenated (95% O2 and 5% CO2) cutting solution for 45 s. The brains were then quickly extracted and placed in the same cutting solution containing the following (in mM): 2 KCl, 7 MgCl2, 0.5 CaCl2, 1.2 NaH2PO4, 26 NaHCO3, 11 D-glucose, and 250 sucrose. Horizontal midbrain slices (200 μm) containing the lateral VTA were obtained using a vibrating microtome (Leica). Slices were incubated for 30 min at 33–34°C with carboxygenated artificial cerebrospinal fluid (ACSF) that also contained the NMDA receptor antagonist MK-801 (10 μM). The ACSF solution contained (in mM): 126 NaCl, 2.5 KCl, 1.2 MgCl2, 2.4 CaCl2, 1.2 NaH2PO4, 21.4 NaHCO3, and 11.1 D-glucose. Slices were then left to stabilize at room temperature for at least 30 min.

Slices were placed in a recording chamber attached to an upright microscope (Nikon Instruments) and maintained at 33–34°C with ACSF perfused at a rate of 2 ml/min. VTA DA-ergic neurons were visually identified based on their location in relation to the midline, third cranial nerve, and the medial terminal nucleus of the accessory optic tract. Neurons were further identified physiologically by the presence of spontaneous pacemaker firing (1–5 Hz) with wide extracellular waveforms (>1.1 ms) and the presence of an outward potassium current (*I*_*A*_), and a hyperpolarization-activated current (*I*H) of >100 pA. Recording pipettes (2.4–2.7 MΩ resistance) were constructed from thin wall capillaries (World Precision Instruments) with a PC-100 puller (Narishige International). Whole-cell recordings were obtained using an internal solution containing the following (in mM): 100 K-gluconate, 20 NaCl, 1.5 MgCl2, 10 HEPES-K, 2 ATP, 0.4 GTP, and 0.025 EGTA, pH 7.35–7.40, 269 –272 mOsm. *Drugs:* The voltage was held at −55 mV in the presence of the fast transmitter receptor antagonists picrotoxin (100 μM, GABAA; TCI) and DNQX (10 μM, AMPA; Sigma Aldrich). A minimum of 5 min of stable baseline was obtained before bath perfusion of NT_8–13_ (300 nM; Sigma Aldrich) for 5 min, followed by at least 10 min of washout.

### Diet tracking and weekly weighing

*Developmental Model*: At 4 weeks of age, study mice were individually housed with *ad libitum* access to water and chow (Harlan Teklad #7913) or 45% high-fat diet (HFD, Research Diets D12451). Food weight and body weight were measured once per week for *DATR1^Null^*, *NtsR1*^flox/flox^*, DAT*^Cre^**, and *Wt* littermates for 12 wks, until mice were 16 wk of age.

*Adult Model*: *NtsR1^flox/flox^* mice and *Wt* littermates that underwent surgery at ∼8 weeks (described below) were housed individually after surgery and kept on *ad libitum* chow (Harlan Teklad #7913) for 8 weeks and then were switched to *ad libitum* 45% high-fat diet (HFD, Research Diets D12451) for the duration of experiments, unless otherwise specified. Diet weight and body weight were measured once per week starting 2 weeks after surgery and finishing 8 weeks after HFD was given.

### Surgery

*NtsR1^flox/flox^* mice and *Wt* littermates (8–12 weeks) were anesthetized (isoflurane/oxygen mixture, 2–4%) and given analgesic (Meloxicam, 5 mg/kg) prior to bilateral stereotaxic injection of AAV1.hSyn.H1.eGFP-Cre.WPRE.SV40 (AAV-Cre-GFP, U Penn Vector Core) or AAV-GFP into the VTA (100 nL per side, A/P: –3.2, M/L: ± 0.48, D/V: –4.65) as per the mouse brain atlas of Franklin and Paxinos (Franklin and Paxinos, 2008). Brains were examined *via post-hoc* immunostaining for GFP and TH (described below) to verify targeting and GFP expression in VTA TH + neurons. Data from injected mice were excluded if GFP-expressing soma were not confined to the VTA.

### Metabolic analysis

Mice were analyzed in PhenoMaster metabolic cages (TSE Systems) that continuously monitored food and water intake, locomotor activity, wheel running, and metabolic parameters (VO2, respiratory exchange ratio [RER], and energy expenditure). Mice were acclimated in cages for 48 h prior to testing day. Body weight was measured before mice were placed in TSE cages. Ambient temperature was maintained at 20–23°C and airflow rate was adjusted to maintain an oxygen differential around 0.3% at resting conditions. Developmental model mice were tested after 16 wks on chow diet. Adult model mice were analyzed at the end of 8 wks on chow and then again at the end of 8 wks on HFD.

### Fasting induced re-feeding

Chow or HFD was removed from home cages at ∼5PM and mice were given a clean cage bottom. Mice had *ad libitum* access to water during food-deprivation. The following morning at ∼9AM, fasted mice were given back chow or HFD pellets. Food intake and body weight were measured 1.5, 12, and 24 h after food was restored. Developmental model mice were tested after 16 wks on chow diet. Adult model mice were analyzed at the end of 8 wks on chow diet and then again at the end of 8 wks on HFD.

### Operant testing

Mice were trained to nose-poke (active hole randomized) for unflavored 20 mg sucrose pellets (TestDiet 1811555) in operant-responding chambers (Med Associates) as previously described (Sharma et al., 2012; Woodworth et al., 2017a). *Training*: Mice were food restricted to 85% of their body weight during FR1 training sessions, which occurred over 10–16 consecutive days. Each FR1 training session was terminated after 1 h or when the mouse had earned 50 reinforcers. Once mice achieved 75% response accuracy with ≥20 reinforcers earned on 3 consecutive days of FR1 training, they were switched to *ad libitum* chow and trained on an FR5 schedule for 3 consecutive days. Mice that failed to reach FR1 criteria after 16 days were removed from the study. *Progressive Ratio (PR) Testing:* After FR5 testing mice were subject to a progressive ratio (PR) schedule where PR = [5e(R^*0.2^)]−5 with R = number of food reinforcers earned + 1. The PR breakpoint was recorded as the highest response requirement completed for each 1 h test session. Mice were tested until they achieved stable PR, defined as <10% variation in rewards earned over 3 consecutive sessions. To determine if hunger alters operant responding, mice were fasted overnight, and then tested on the PR schedule.

### Open field and amphetamine test

Mice were placed in a quiet room under red lights for an hour before placement in open field boxes. For open field, locomotor activity was measured and analyzed for 30 min using a digital CCD camera and video-tracking software (Clever Sys) (Eagle et al., 2015). For amphetamine (AMPH) trials, developmental model mice were injected with PBS ∼8 min after being placed in the box and then again with AMPH at the ∼40-min mark. Developmental model mice were tracked for a total of 70 min. Adult HFD model mice were treated with PBS immediately before being placed in the boxes, and then with AMPH at the ∼30-min mark. Adult HFD model mice were tracked for a total of 90 min.

### Nestlet shredding

Mice were placed in a quiet room in a clean home cage and any nestlet material or enrichment was removed overnight. On testing day (light cycle), food and water were removed, and a pre-weighed, new cotton nestlet was added to the cage. After 30 min, intact remnants of the nestlet were removed from the cage and weighed. If the nestlet was found to be wet, it was air dried overnight and weighed the following day.

### Marble burying

Mice were placed in a quiet room in home cage and any nestlet material or enrichment was removed overnight. On testing day (light cycle), mice were placed in the middle of a clean cage with 10 evenly dispersed marbles on top of the bedding. After 30 min mice were returned to their home cage. Photos taken before and after the test were used to determine the number and percentage of marbles buried. Photos were assigned new, non-genotype descriptive ID’s to facilitate blinded data entry. A marble was counted as buried if 2/3 of the marble was covered.

### RNAScope

Mice were intracardially perfused with 10% formalin (*n* = 3 per genotype/viral injection), then brains were extracted, postfixed in 10% formalin overnight at 4°C and dehydrated with 30% sucrose in PBS for 2–3 days. Brains were then coronally sectioned (30 μm) for RNAScope, as per (Woodworth et al., 2018). Three free floating sections of the VTA per mouse were selected for application of RNAScope single-plex assay (catalog #322360, Advanced Cell Diagnostics) per the manufacturer’s protocol. Sections were washed in 1X PBS and incubated in Pretreatment 1 (H_2_O_2_) at room temperature (RT) until bubbling stopped (45–60 min) followed by 0.5X PBS wash and mounting on positively charged slides. Following washing in dH_2_O and drying at 60°C oven, sections were incubated in 1X Pretreatment 2 (Target Retrieval Agent) for 5–10 min at 99–104°C and then washed with dH_2_O, dried at RT, dipped in 100% EtOH and air dried. Next, they were incubated in Pretreatment III solution (Protease Plus) for 15 min at 40°C, followed by dH_2_O wash. Sections were then incubated in NtsR1 (cat #: 422411, Advanced Cell Diagnostics) probes for 2 h in a humidified oven at 40°C. After amplification steps (Amp1-6), hybridization was visualized by application of Fast-Red-A and Red-B (60:1) for 15–20 min. Finally, after washing Fast-Red solution, slides were dehydrated by dipping into xylene and cover-slipped with antifade mounting agent.

### Perfusion and immunofluorescence

Mice received a lethal *i.p.* dose of pentobarbital (Fatal Plus, Vortech) followed by transcardial perfusion with 0.2M PBS (pH 7.4) and then 10% neutral-buffered formalin (Fisher Scientific). Brains were removed, postfixed, and coronally sectioned into four series of 30 μm sections using a freezing microtome (Leica). Free-floating sections were exposed to primary antibodies including chicken-GFP (1:2000, Abcam, ab13970) and mouse anti-TH (1:1000, Millipore, AB_2201528) followed by species-specific secondary antibodies conjugated to Alexa-568 (1:200, LifeTech, AB_2534017) or Alexa-488 (1:200, Jackson ImmunoResearch, 703-545-155). Sections mounted onto slides were analyzed *via* an Olympus BX53 fluorescence microscope with FITC and Texas Red filters. Images were collected using Cell Sens software and a Qi-Click 12 Bit cooled camera and analyzed using Photoshop (Adobe). Masks applied in Photoshop to enhance brightness and/or contrast were applied uniformly to all samples.

### Statistics

Unpaired Student’s *t*-tests and ordinary 2-way ANOVA with Sidak post-test were calculated using Prism 7 (GraphPad). A *p*-value of <0.05 was considered statistically significant. *p < 0.05, ^**^p < 0.01, ^***^p < 0.001, ^****^p < 0.0001.

## Results

### Characterization of NtsR1*^flox/flox^* mice

**FIGURE 1:**
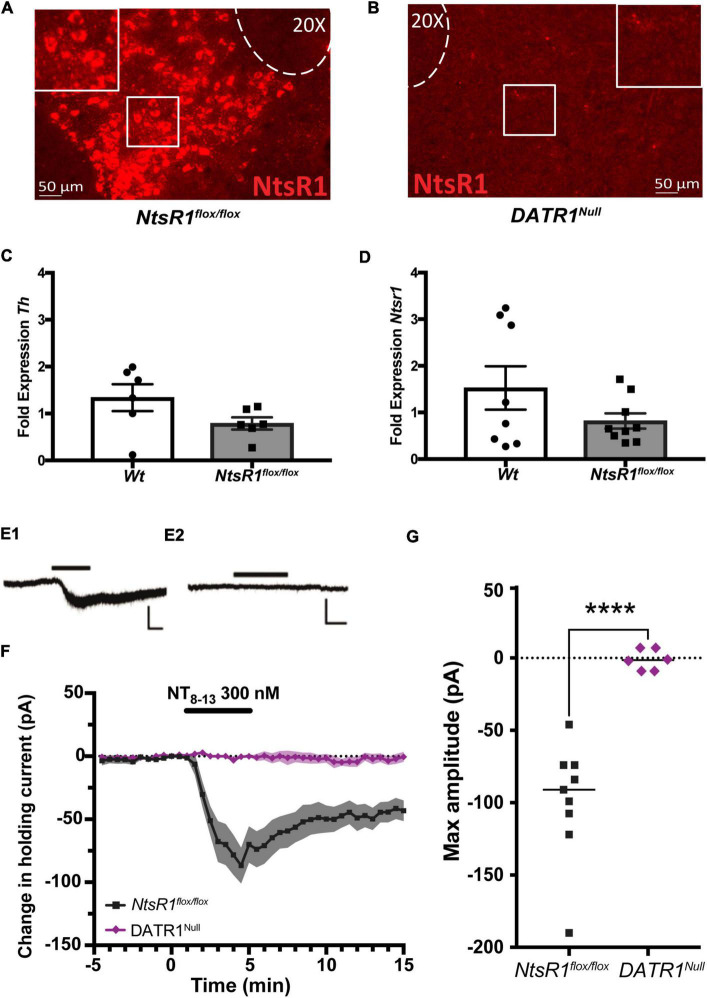
Characterization of NtsR1*^flox/flox^* mice and developmental deletion. **(A,B)** RNAScope analysis of *Ntsr1* mRNA (red) confirms **(A)** intact *Ntsr1* expression in the VTA of *NtsR1^flox/flox^* mice but **(B)** absence of *Ntsr1* in *DATR1^Null^* mice, indicative of Cre-mediated NtsR1 deletion. **(C,D)** Analysis of VTA tissue from *NtsR1^flox/flox^* and *Wt* littermates for fold difference in gene expression of panel **(C)** *Tyrosine hydroxylase (Th)* and **(D)** *Ntsr1*. Graphs depict the fold change in gene of interest normalized to *Gapdh* ± SEM. **(E1,2)** Representative recording traces of VTA dopaminergic neurons from *NtsR1^flox/flox^* **(E1)** and *DATR1^Null^* **(E2)** during bath perfusion of the active peptide fragment NT_8–13_ (5 min, 300 nM; horizontal black line). Scale bars: 100 pA, 2 min. **(F)** Summary data showing time course of change in holding current produced by NT_8–13_ (*n* = 9 *NtsR1^flox/flox^* and 6 *DATR1^Null^* cells). Graph depicts the mean change in holding current ± SEM. **(G)** Maximum change from baseline in holding current for individual cells. Graph depicts mean maximum amplitude. ****p < 0.0001 *via* unpaired Student’s *t*-test.

We first assessed whether our strategy can developmentally and conditionally delete NtsR1 from DA neurons by crossing *NtsR1*^flox/flox^** and *DAT^Cre^* mice. Progeny with both *DAT^Cre^* and *NtsR1^flox/flox^* are referred to as *DATR1^Null^* mice and should exhibit early developmental Cre-mediated deletion of NtsR1 from all DAT-expressing neurons. In the absence of *DAT^Cre^*, *NtsR1^flox/flox^* mice exhibited robust *Ntsr1* mRNA expression in the VTA (Figure 1A), consistent with the reported expression of NtsR1 in this brain region (Palacios et al., 1988; Lepee-Lorgeoux et al., 1999; Woodworth et al., 2017a,^2018^; Perez-Bonilla et al., 2021). In contrast, *NtsR1* expression was absent from the VTA of *DATR1^Null^* mice, confirming successful *DAT^Cre^*-mediated deletion of *Ntsr1*, and that this strategy can be used to developmentally delete NtsR1 from Cre-expressing neurons (Figure 1B). Next, we investigated whether introducing loxP sites flanking NtsR1 exon 1 impacts DA neurons by measuring gene expression in VTA from Wt controls and *NtsR1^flox/flox^* mice. In the absence of Cre expression, *NtsR1^flox/flox^* mice should retain intact *NtsR1* expression comparable to the Wt mice. The lack of significant differences in fold expression of *Th* or *Ntsr1* (Figures 1C,D) suggests that loxP insertion itself does not disrupt baseline gene expression of VTA DA neurons. We then examined whether Cre-mediated deletion of NtsR1 from *NtsR1^flox/flox^* mice functionally impacted the response of VTA NtsR1 neurons to Nts using VTA-containing slices from *NtsR1^flox/flox^* (with intact NtsR1) and *DATR1^Null^* (developmental deleted) mice. Bath application of Nts produced a significant inward shift in holding current in VTA DA neurons from *NtsR1^flox/flox^* mice with intact NtsR1, but these Nts-mediated responses were absent in VTA DA neurons from *DATR1^Null^* mice (Figures 1E–G). These data support the functional loss of NtsR1 from *DATR1^Null^* mice and suggest that these mice can be used to assess the impact of deleting NtsR1 from DA neurons.

### Developmental deletion of neurotensin receptor-1 from dopamine neurons in normal weight, chow-fed mice

**FIGURE 2:**
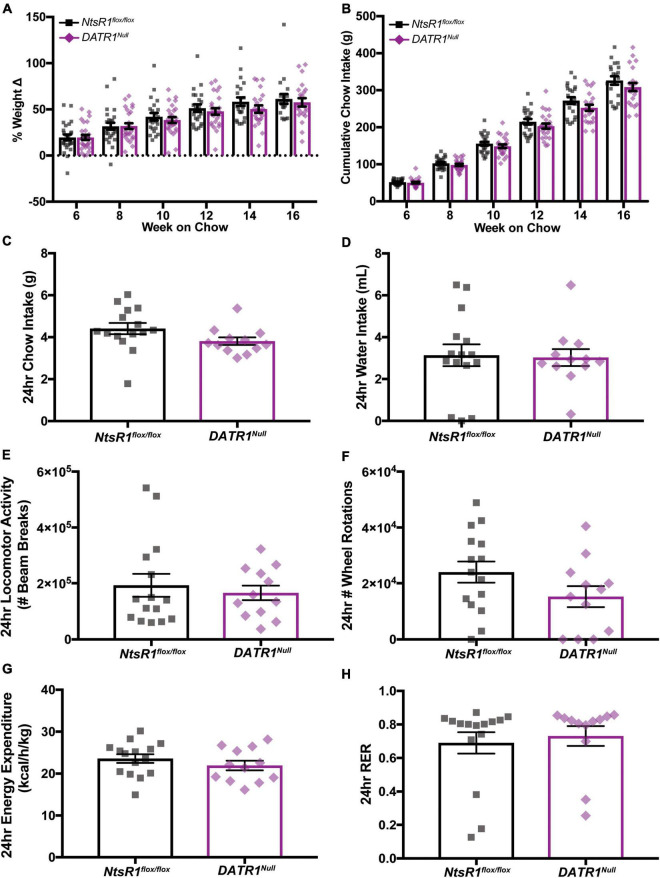
Developmental deletion of NtsR1 from DA neurons has no impact on body weight. **(A,B)** Chow fed *NtsR1^flox/flox^* (Controls with intact NtsR1) and *DATR1^Null^* mice (with developmental NtsR1 deletion from DA neurons) were individually housed at 4 weeks old and analyzed in home cages. Body and chow weight was measured weekly for 12 weeks. **(A)** % weight change every 2 weeks from week 6 to 16. **(B)** Cumulative chow intake every 2 weeks from week 6 to 16. Both *NtsR1^flox/flox^* and *DATR1^Null^* mice gain weight and consume chow indistinctly from each other. Data represent mean ± SEM analyzed *via* ordinary 2Way-ANOVA with Sidak’s correction. *n* = 12–13. **(C–H)** Chow fed *NtsR1^flox/flox^* and *DATR1^Null^* mice were analyzed in metabolic cages. Data was pooled for a ∼24 h period that contains a light and dark cycle. Developmental deletion of NtsR1 from DA neurons did not alter **(C)** chow intake, **(D)** water intake, **(E)** ambulatory locomotor activity, **(F)** number of wheel rotations, **(G)** energy expenditure or **(H)** respiratory exchange ratio (RER). Data represent mean ± SEM. Body weight was analyzed with two-tailed unpaired *t*-test (*n* = 12–13).

We then examined how developmental deletion of NtsR1 from DAT-expressing neurons (e.g., DA neurons) impacts body weight. To do this, we measured the weekly body weight and food intake of chow fed *NtsR1^flox/flox^* and *DATR1^Null^* mice with developmental deletion of NtsR1 from DA neurons while in their individual home cages (Figures 2A,B). Deleting NtsR1 from all DA expressing neurons did not alter body weight or chow intake over 16 weeks (Figures 2A,B). We also analyzed *NtsR1^flox/flox^* and *DATR1^Null^* mice *via* metabolic chambers (Figures 2C–H), but observed no differences between groups in chow intake, water intake, ambulatory locomotor activity, wheel rotations, energy expenditure or RER over 24 h of measurement (Figures 2C–H). Neither were any group differences noted across the light/dark cycle (data not shown). These data suggest that developmental deletion of NtsR1 from DA neurons is not sufficient to overtly disrupt metabolic phenotype.

### Developmental deletion of neurotensin receptor-1 from dopamine neurons does not increase dopamine-dependent food consumption

**FIGURE 3:**
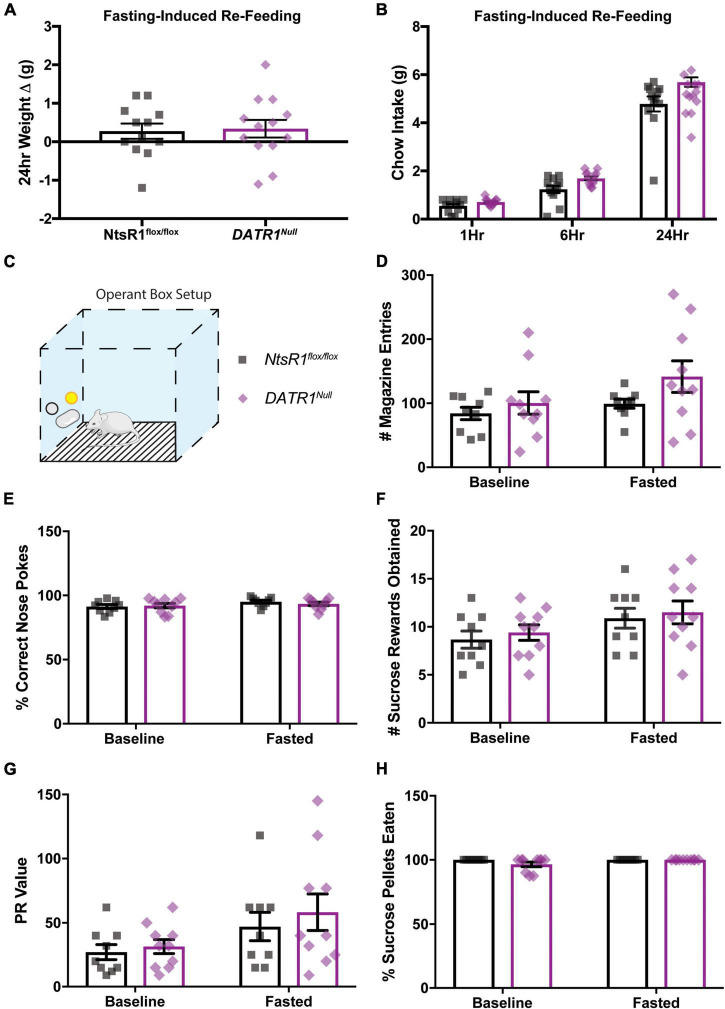
Developmental deletion of NtsR1 from DA neurons does not alter fasting-induced or operant feeding response. *NtsR1^flox/flox^* and *DATR1^Null^* mice (with developmental deletion of NtsR1 from DA neurons) were fasted overnight. Mice were re-fed the next day with normal chow diet and tested in operant boxes to assess whether developmental NtsR1 deletion decreases DA-mediated food intake. **(A)** Change in body weight 24 h after food was restored. **(B)** Chow intake measured 1, 6, and 24 h after chow was restored. After fasting-induced re-feeding studies, mice were tested in operant boxes. **(C)** Depiction of operant box setup. The position of the correct nose-poke (yellow) was counterbalanced between mice. *NtsR1^flox/flox^* and *DATR1^Null^* were trained to nose poke for sucrose rewards, then were tested on a PR schedule for operant responding during baseline (control, *ad libitum* chow) and fasted (hungry) conditions. No differences between groups were observed in panel **(D)** magazine entries (where sucrose pellet is deposited), **(E)** correct nose pokes, **(F)** number of sucrose pellets obtained, **(G)** PR value (last ratio obtained), and **(H)** percentage of sucrose pellets eaten. Data were analyzed by Ordinary One-Way ANOVA with Sidak post-tests. Graphs represent mean ± SEM (*n* = 9–13).

Next, we examined whether developmental deletion of NtsR1 from DA neurons altered the ability of *DATR1^Null^* mice to coordinate ingestive behavior. *NtsR1^flox/flox^* control mice and *DATR1^Null^* mice exhibited similar levels of fasting-induced refeeding and body weight gain, suggesting normal responses to hunger (Figures 3A,B). Given the important role of the DA system in motivated responding for palatable food rewards, we then examined whether developmental deletion impacted operant responding for sucrose, which is a DA-dependent behavior (Figure 3C). Further, we tested mice that were sated (baseline) and after overnight fasting to assess whether hunger altered motivated responding for sucrose. *NtsR1^flox/flox^* control mice and *DATR1^Null^* mice had comparable numbers of magazine entries, percentages of correct nose pokes, earned sucrose reinforcers, PR values and ate a similar percentage of sucrose pellets across baseline and fasted conditions (Figures 3D–H). Overall, these results suggest that developmental deletion of NtsR1 from DA neurons doesn’t impair coordinated feeding responses to hunger nor goal-directed behavior needed to obtain food.

### Impact of developmental neurotensin receptor-1 deletion from dopamine neurons on locomotor activity and anxiety

**FIGURE 4:**
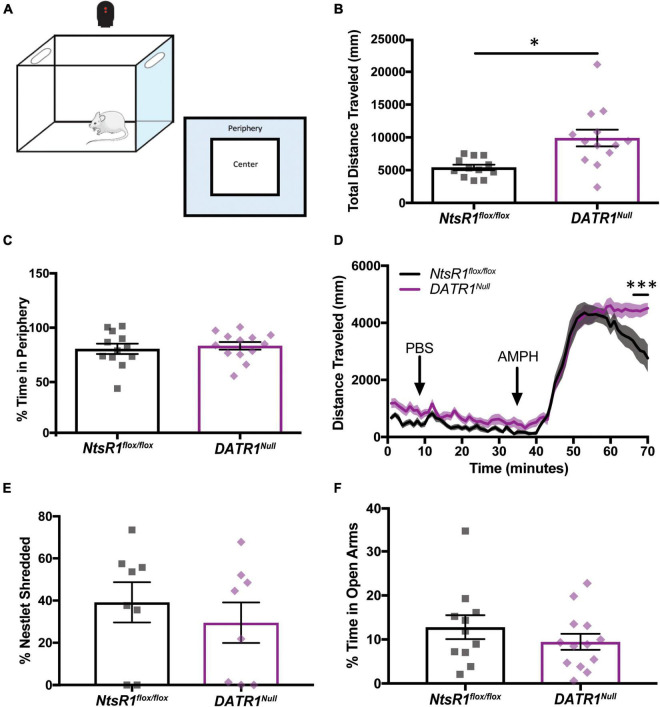
Developmental deletion of NtsR1 increases DA-dependent locomotor activity without inducing anxiety. *NtsR1^flox/flox^* and *DATR1^Null^* mice were placed in open field boxes and assessed for exploratory behavior and locomotor activity **(A)**. In panels **(B,C)**, mice were left to explore box for 30 min. Graphs depict the last 10 min of collected data. In panel **(D)**, mice were left to explore the box for 30 min after PBS injection, and for 30 min after amphetamine (AMPH) treatment. **(B)** Total distance traveled under baseline condition (no injection). **(C)** % time spent in box periphery under baseline condition. These data suggest that developmental deletion of NtsR1 from DA neurons increases the quantity of exploratory behavior but does not alter its quality. **(D)** Distance traveled before, during and after PBS and AMPH treatment, binned in 1 min intervals. *DATR1^Null^* mice had a significantly prolonged response to AMPH compared to *NtsR1^flox/flox^* mice. To assess whether *DATR1^Null^* mice engaged in anxiety-like behaviors, they were given a nestlet to shred for 30 min and placed in the Elevated Plus Maze (EPM) for 5 min. Developmental deletion of NtsR1 did not significantly alter **(E)** % of nestlet shredding or **(F)** % time spent in open EPM arms. Open field, nestlet shredded and EPM data represent mean ± SEM analyzed *via* unpaired *t*-test (*n* = 8–13). AMPH data represent mean ± SEM analyzed *via* ordinary 2-way ANOVA with Sidak post-tests (*n* = 12–13). *p < 0.05, ***p < 0.001.

We used open field chambers to determine whether developmental deletion of NtsR1 from DA neurons impacts DA-dependent locomotor activity and anxiety behaviors (Figures 4A–F). When placed in the novel open field environment, *DATR1^Null^* mice traveled more distance compared to *NtsR1^flox/flox^* littermates (Figure 4B), indicative of increased exploratory behavior. Since *DATR1^Null^* mice and *NtsR1^flox/flox^* controls spent comparable percentage of time in the periphery, it suggests that the developmental deletion of NtsR1 does not invoke anxiety-like movement (Figure 4C). Notably, constitutive NtsR1 knockout mice also exhibit elevated basal and stimulant-induced locomotor activity that is thought to be due to elevated DA signaling (Liang et al., 2010). We therefore examined whether developmental deletion of NtsR1 from DA neurons similarly promotes hyperactivity by measuring amphetamine (AMPH)-induced locomotor activity in *DATR1^Null^* and *NtsR1^flox/flox^* littermates. Importantly, this paradigm is well established to assess the integrity of the mesolimbic DA system. We found that *DATR1^Null^* mice and *NtsR1^flox/flox^* controls exhibited similar locomotor responses to PBS (control) treatment. AMPH injection (4mg/kg *i.p*.) significantly increased locomotor activity in both groups, as expected, but *DATR1^Null^* mice maintained significantly higher AMPH-induced locomotor activity compared to the control mice (Figure 4D). These data indicate that loss of NtsR1 from DAT expressing neurons may enhance DA signaling, similar to compensatory increases in extracellular DA that occur with partial loss of DA neurons (Robinson et al., 1994) or constitutive NtsR1 deletion. Although hyperactivity and excessive DA release have been associated with anxiety, we found no differences in anxiety-like behaviors between *DATR1^Null^* and *NtsR1^flox/flox^* mice as assessed *via* percentage of time spent in the periphery of open field chambers (Figure 4C), nor *via* nestlet shredding or elevated plus maze (EPM) tests (Figures 4E,F).

### Developmental deletion of neurotensin receptor-1 from dopamine neurons protects mice from diet-induced obesity

**FIGURE 5:**
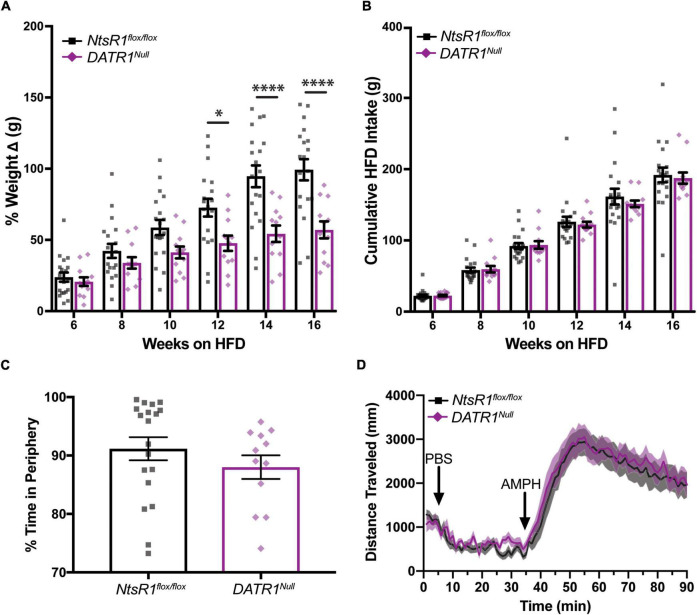
Developmental deletion of NtsR1 partially protects mice from diet-induced obesity. **(A,B)** HFD fed *NtsR1^flox/flox^* and *DATR1^Null^* mice were individually housed and analyzed in home cages. Body and HFD weight was measured weekly. **(A)** % weight change every 2 weeks. HFD fed *DATR1^Null^* have significantly lower weight compared to *NtsR1^flox/flox^* mice on the same diet during weeks 12–16. **(B)** Cumulative HFD intake every 2 weeks. *NtsR1^flox/flox^* and *DATR1^Null^* mice consume similar amounts of HFD. Data represent mean ± SEM analyzed via Ordinary 2-Way-ANOVA with Sidak’s correction. **(C)** % time spent in open field box periphery (30 min). **(D)** Distance traveled before, during and after PBS and AMPH treatment, binned in 1 min intervals. Mice were left to explore the open field box for 30 min after PBS injection, and for 55 min after amphetamine (AMPH) treatment. *n* = 12–20. *p < 0.05, ^****^p < 0.0001.

*NtsR1^flox/flox^* and *DATR1^Null^* mice were given *ad libitum* high fat diet (HFD) in their home cages, which is well established to promote obesity. As expected, both groups of mice gained weight over the course of 16 weeks on HFD. However, the weight gain of *DATR1^Null^* mice was significantly attenuated compared to *NtsR1^flox/flox^* controls from weeks 12–16 (Figure 5A). This protection from developing diet induced obesity was not attributable to reduced caloric intake, since the amount of HFD consumed was similar between both groups (Figure 5B). Neither was the protection from weight gain due to enhanced energy expenditure, as both groups exhibited similar RER, VO_2_ and CO_2_ when analyzed in metabolic cages (>Supplementary Figure 1). *DATR1^Null^* and *NtsR1^flox/flox^* control mice also had similar levels of total open field locomotor activity (data not shown) although they spent less time in periphery. Additionally, *DATR1^Null^* and *NtsR1^flox/flox^* control mice exhibited similar AMPH-induced locomotor activity (Figures 5C,D). These data suggest that developmental deletion of NtsR1 from DA neurons provides some protection from diet-induced weight gain, but it is not mediated *via* behavioral alterations in feeding, movement or energy expenditure.

### Verification of site-specific neurotensin receptor-1 deletion from adult ventral tegmental area neurons

**FIGURE 6:**
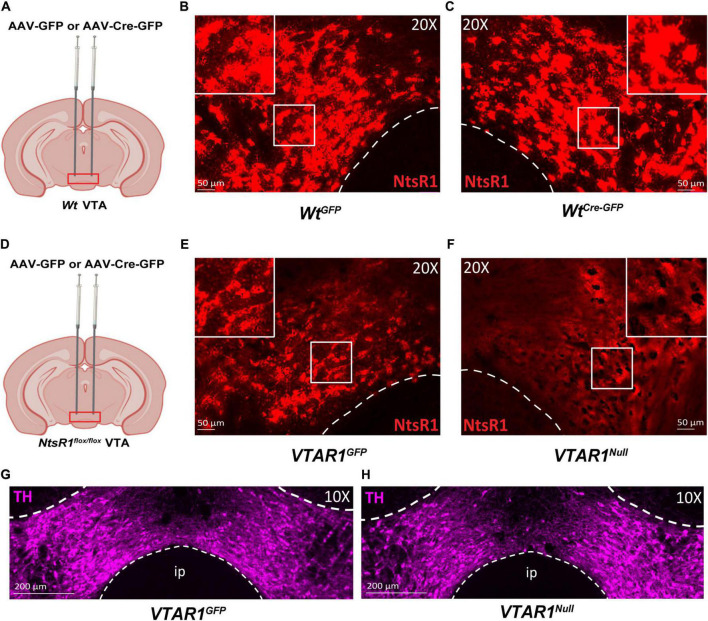
Injection of AAV-Cre-GFP deletes NtsR1 from DA neurons of adult *NtsR1^flox/flox^* mice. **(A–F)** Adult *Wt* mice or *NtsR1^flox/flox^* mice were injected bilaterally in the VTA with either AAV-GFP or AAV-Cre-GFP. VTA tissue from perfused mice was assessed for *Ntsr1* mRNA expression (red) by RNAScope assay. *Wt* mice injected with **(B)** AAV-GFP and **(C)** AAV-Cre-GFP have comparable distribution of *Ntsr1* in the VTA. **(D)** *NtsR1^flox/flox^* mice injected in the VTA with **(E)** AAV-GFP have intact *Ntsr1* mRNA expression but **(F)** it is absent in the VTA of AAV-Cre-GFP injected mice. Distribution of tyrosine hydroxylase (TH, magenta) protein *via* immunofluorescence in panel **(G)** AAV-GFP injected *NtsR1^flox/flox^* mice, referred to as *VTAR1^GFP^*, and AAV-Cre-GFP injected *NtsR1^flox/flox^* mice, referred to as *VTAR1^Null^* mice. Images are representative of *n* = 3 mice per genotype and injection. Scale bars = 50 μm, 200 μm.

Developmental gene deletion often leads to compensatory actions that mask the importance of the gene product, which could have occurred in our developmental deletion of NtsR1 from DA neurons. Furthermore, DA neurons are distributed in several midbrain regions, hence, the method we used to delete NtsR1 from all DA neurons could not confirm the role of NtsR1 specifically *via* the DA neurons of the VTA. We reasoned that selectively inducing Cre in the VTA of adult *NtsR1^flox/flox^* mice would permit adult-onset, VTA-specific deletion of NtsR1 that could be used to reveal the importance of NtsR1 *via* this brain region. To test the utility of this method, we administered bilateral VTA injections of AAV-GFP or AAV-Cre-GFP to *Wt* and *NtsR1^flox/flox^* mice and ∼6 months later analyzed *Ntsr1* mRNA expression by RNAScope (Figures 6A–F). *Wt* mice injected with AAV-GFP (*Wt^GFP^* mice) or AAV-Cre-GFP (*Wt^Cre–GFP^* mice) had similar levels of *Ntsr1* expression in the VTA, confirming these methods did not delete *Ntsr1* (Figures 6A–C). *NtsR1^flox/flox^* mice injected with AAV-GFP also had intact *Ntsr1* (*VTAR1^GFP^* mice, Figure 6E). However, *Ntsr1* expression was visibly absent in *NtsR1^flox/flox^* mice that received AAV-Cre-GFP injections into the VTA, confirming sustained *Ntsr1* deletion (Figure 6F). By contrast, both groups had comparable distributions of TH-expressing neurons (DA neurons) in the VTA (Figure 6G), suggesting intact DA systems. Taken together, these data confirm that this viral-mediated approach can site-specifically delete NtsR1 in adult mice but does not cause overt disruptions in the distribution of DA neurons.

### Ventral tegmental area-specific deletion of neurotensin receptor-1 from adult mice decreases body weight and dopamine-dependent chow intake

**FIGURE 7:**
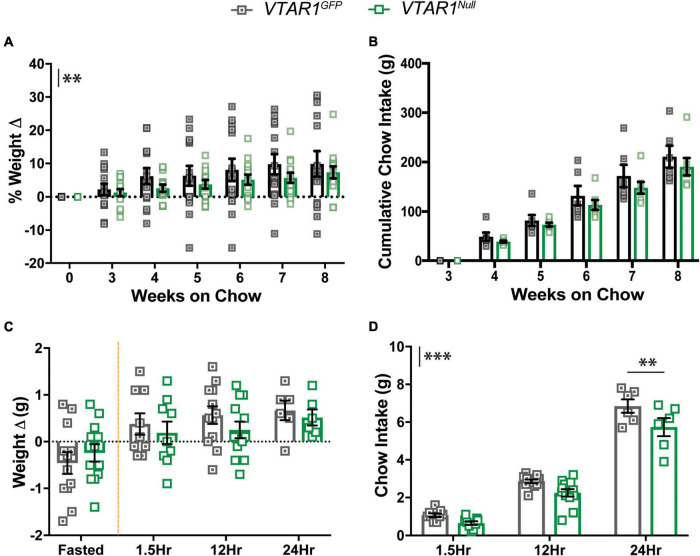
Ventral tegmental area specific NtsR1 deletion in adulthood decreases body weight and DA-dependent chow intake. *VTAR1^GFP^* and *VTAR1^Null^* mice were individually housed immediately after surgery and their chow and body weight was measured weekly until the 8-week mark after surgery. **(A)** % weight change from surgery day to week 8 on chow and **(B)** cumulative chow intake. *VTAR1^Null^* mice gain less weight compared to *VTAR1^GFP^* mice. Data represent mean ± SEM analyzed *via* ordinary 2Way-ANOVA with Sidak’s correction. *n* = 6–13. *VTAR1^GFP^* and *VTAR1^Null^* mice were fasted overnight and then re-fed the next day with normal chow to assess whether adult NtsR1 deletion decreases DA-mediated food intake. Weight was measured ∼15 h after mice were fasted and 1.5, 12, and 24 h after food was restored. **(C)** Weight change before and after normal chow was restored. **(D)** Chow intake after chow was restored. *VTAR1^Null^* mice on normal chow diet gained weight similar to *VTAR1^GFP^* mice, but their chow intake was significantly reduced at 24 h. Data represent mean ± SEM analyzed *via* ordinary 2Way-ANOVA with Sidak’s correction for multiple comparisons. *n* = 12. Mark next to the *y*-axis in panels **(A–D)** indicates overall significant differences between genotypes. **p < 0.01, ***p < 0.001.

To determine the impact of deleting NtsR1 from established VTA DA neurons we measured the body weight and food intake of *VTAR1^GFP^* mice (with intact NtsR1 in VTA DA neurons) and *VTAR1^Null^* mice (lacking NtsR1 in VTA DA neurons) on a normal chow diet (Figures 7A,B). Adult-onset deletion of NtsR1 from VTA neurons resulted in overall lower body weight (Figure 7A; *y* axis asterisks indicate overall virus effect) but did not reduce overall cumulative chow intake (Figure 7B). By contrast, no differences in body weight or feeding were observed in *Wt* mice injected with AAV-Cre-GFP or AAV-GFP (>Supplementary Figures 2A,B). These data suggest that NtsR1 contributes to modulating body weight, but it is not necessarily required to regulate homeostatic food intake. Given that food restriction enhances DA release in response to food rewards, and since NtsR1 expressing neurons are a subpopulation of all VTA DA neurons, we reasoned that deleting NtsR1 from adult VTA DA neurons might attenuate fasting-induced refeeding. Overnight fasted *VTAR1^GFP^* and *VTAR1^Null^* mice regained similar amounts of weight after re-feeding, but *VTAR1^Null^* mice consumed significantly less chow overall, particularly 24 h after fasting compared to *VTAR1^GFP^* controls (Figures 7C,D). These effects were not observed in *Wt* mice injected with AAV-Cre-GFP or AAV-GFP (>Supplementary Figures 3C,D), confirming that Cre-mediated recombination is necessary for the alterations in body weight and feeding behavior.

### Neurotensin receptor-1 deletion from adult ventral tegmental area dopamine neurons does not alter dopamine-dependent locomotor activity or anxiety behaviors

**FIGURE 8:**
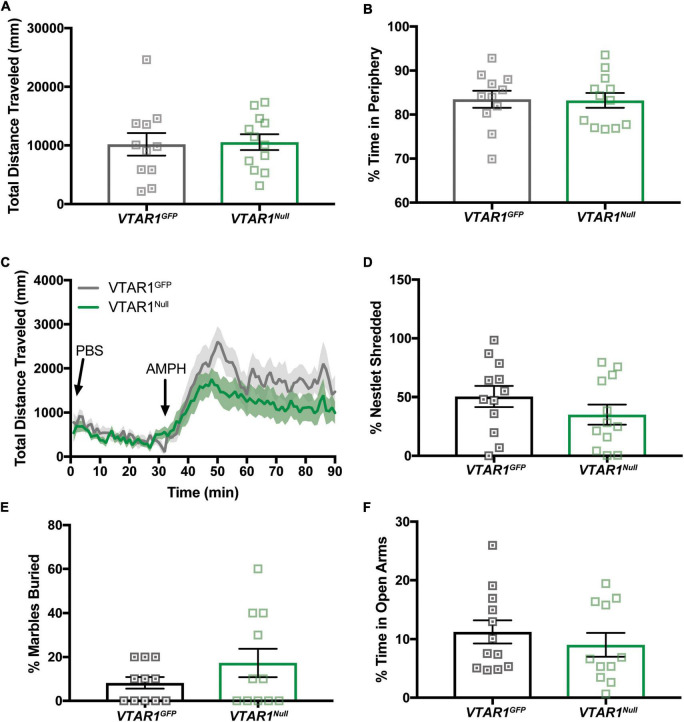
Specific VTA NtsR1 deletion does not alter DA-dependent locomotor activity or anxiety behaviors. *VTAR1^GFP^* and *VTAR1^Null^* mice were placed in open field boxes and videotracked to assess exploratory behavior and locomotor activity. In panels **(A,B)**, mice were left to explore box for 30mins, bars represent last 15 min of data collected and analyzed. In panel **(C)**, mice were left to explore the box for 30 min after PBS injection, and for 30 min after amphetamine (AMPH) treatment. **(A)** Total distance traveled under baseline condition (no injection). **(B)** % time spent in box periphery under baseline condition. **(C)** Total distance traveled before, during and after PBS and AMPH treatment. *VTAR1^Null^* mice did not have a significantly prolonged response to AMPH compared to *VTAR1^GFP^* mice. To assess whether *VTAR1^Null^* mice engaged in anxiety-like behaviors, they were given a nestlet to shred and marbles to bury for 30 min and placed in the Elevated Plus Maze (EPM) for 5 min. Deletion of NtsR1 specifically from the VTA in adulthood did not significantly alter **(D)** % of nestlet shredding, **(E)** % of marbles buried, or **(F)** % time spent in open EPM arms. Open field, nestlet shredded, marbles buried and EPM data represent mean ± SEM analyzed *via* unpaired *t*-test. *n* = 8–13. AMPH data represent mean ± SEM analyzed *via* ordinary 2-way ANOVA with Sidak post-tests. *n* = 12–13.

We next used open field chambers to assess whether adult-onset deletion of NtsR1 from VTA DA neurons altered DA-dependent locomotor activity and anxiety behaviors (Figures 8A–F). *Wt* mice injected with AAV-Cre-GFP or AAV-GFP exhibited comparable amounts of open field exploration, AMPH-induced locomotor activity, nestlet shredding, marble burying and EPM performance (>Supplementary Figures 4A–F). The *VTAR1^Null^* and *VTAR1^GFP^* control mice performed comparably to each other in these tests (Figures 8A–F). These data indicate that adult-onset deletion of NtsR1 from VTA DA neurons does not impair locomotor or anxiety responding in normal weight, chow-fed mice.

### Deletion of neurotensin receptor-1 from adult dopamine neurons in obesity limits weight gain and dopamine-dependent food intake

**FIGURE 9:**
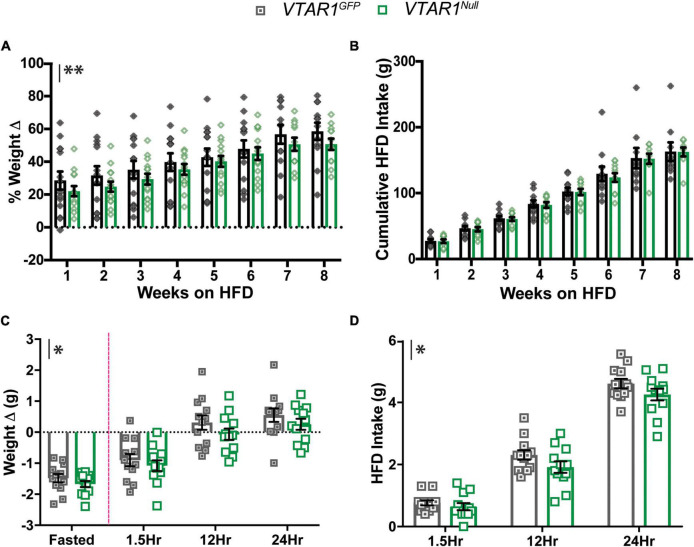
Adult VTA NtsR1 deletion in obesity decreases body weight and DA-dependent HFD intake. *VTAR1^GFP^* and *VTAR1^Null^* mice were switched to HFD after 8 weeks of normal chow. Body and HFD weight were measured weekly for 8 weeks of HFD. **(A)** % Weight change on HFD and **(B)** cumulative HFD Intake. *VTAR1^Null^* mice both have lower body weights on HFD than *VTAR1^GFP^* mice on same diet. However, HFD intake is not significantly different between HFD groups. Data represent mean ± SEM analyzed *via* ordinary 2Way-ANOVA with Sidak’s correction. *n* = 12–13. HFD *VTAR1^GFP^* and *VTAR1^Null^* mice were fasted overnight and then re-fed the next day with HFD to assess whether adult NtsR1 deletion decreases DA-mediated HFD intake. Weight was measured ∼15 h after mice were fasted and 1.5, 12, and 24 h after HFD was restored. **(C)** Weight change before and after HFD was restored. **(D)** HFD intake after chow was restored. *VTAR1^Null^* mice on HFD gained less weight compared to *VTAR1^GFP^* mice, and their HFD intake was overall significantly reduced. Data represent mean ± SEM analyzed *via* ordinary 2Way-ANOVA with Sidak’s correction for multiple comparisons. *n* = 12–13. Mark next to the *y*-axis in panels **(A–D)** indicates overall significant differences between genotypes. *p < 0.05, **p < 0.01.

Lastly, we examined whether deletion of NtsR1 from adult VTA DA neurons impacts the susceptibility to develop diet-induced obesity. After completing assessment on chow diet, *VTAR1^GFP^* and *VTAR1^Null^* mice were switched to *ad libitum* HFD in their individual home cages over 8 wks (Figures 9A,B). Both groups gained weight on HFD, but the *VTAR1^Null^* mice lacking NtsR1 in VTA neurons maintained modestly reduced body weight compared to controls (Figure 9A; *y* axis asterisks indicate overall virus effect). However, VTA specific deletion of NtsR1 did not reduce overall cumulative HFD intake (Figure 9B). These data suggest that NtsR1 in the established VTA contributes to body weight regulation, but independent of food intake, since loss of VTA NtsR1 expression had no impact on *ad libitum* HFD feeding. Lastly, we assessed whether obese mice with adult-deletion of NtsR1 from VTA DA neurons were able to appropriately coordinate energy need and feeding *via* testing their fasting-induced refeeding response. Overnight fasting caused *VTAR1^GFP^* and *VTAR1^Null^* mice to lose weight, and then to consume HFD when it was returned the following morning and regain weight. However, *VTAR1^Null^* mice had modest reductions in post-fasting weight regain and HFD consumed compared to *VTAR1^GFP^* control mice with intact NtsR1 HFD (Figures 9C,D). These effects were not observed in *Wt* mice on HFD injected with AAV-Cre-GFP or AAV-GFP (>Supplementary Figures 2A–D), indicating that viral injection itself does not impact energy balance. Overall, these data support that Cre-lox mediated adult-onset deletion of NtsR1 from VTA DA neurons modestly protects mice from diet-induced obesity and hunger-mediated feeding.

## Discussion

In this study we examined whether NtsR1 expression in DA neurons is necessary for energy balance in the face of normal and obesity-promoting diets. We also examined the temporal importance of NtsR1 expression for energy balance *via* deleting NtsR1 from DA neurons during early development vs. from adult DA neurons of the VTA. Our data show that developmental deletion of NtsR1 from DA neurons has little impact on normal body weight but is protective in the face of diet-induced obesity, though this is not attributable to alterations in feeding or energy expenditure. VTA-specific deletion of NtsR1 in adult mice modestly reduced body weight without disrupting *ad libitum* feeding or locomotor activity. Yet, when challenged with hunger, mice with adult-deletion of VTA NtsR1 had impairments in coordinating refeeding responses. Taken together, developmental loss of NtsR1 from DA neurons protects from obesity *via* yet unknown mechanisms independent of feeding and locomotor activity, while loss of NtsR1 expression from adult VTA DA neurons also impedes normal coordination of energy status and feeding.

We generated NtsR1 “floxed” mice to permit conditional deletion of NtsR1. We used the *NtsR1^flox/flox^* mice to explore the requirement for NtsR1 in DA neurons and whether it may differ throughout lifespan, but the line can be used to delete NtsR1 in any Cre-expressing cell type of interest. A caveat of this study is that the *NtsR1^flox/flox^* mice may not be physiologically identical to *Wt* mice. Indeed, this is a concern of any genetically modified mouse line, as introducing genetic alterations into genomic DNA can influence expression of adjacent genes and cell function (Harno et al., 2013). Despite this, it has not yet become common practice to assess gene expression in Cre or flox lines, and it is likely that many lines may in fact differ from *Wt* mice in ways that have yet to be appreciated. Here we investigated the potential impact of introducing loxP sites flanking exon 1 of *Ntsr1* and found that this did not significantly alter NtsR1 gene expression compared to *Wt* littermates. Neither did we observe any alteration in the distribution of TH-expressing neurons in the VTA of *NtsR1^flox/flox^* mice from controls, suggesting that the genetic manipulation did not cause generalized disruption of the neurons. However, to control for the possibility of any altered expression or function in these mice, we only intercompared behaviors of mice on the *NtsR1^flox/flox^* background. This conservative strategy allowed us to, for the first time, assess the impact of deleting NtsR1 from DA neurons. Going forward, *NtsR1^flox/flox^* mice will be useful to parse the role of NtsR1 *via* distributed cell types throughout the brain and body, and to reveal how and where NtsR1 mediates the vast array of physiology that has been attributed to it.

We found that developmental NtsR1 deletion from DA neurons in chow-fed mice did not alter food intake, locomotor activity, or body weight. Interestingly, deletion did result in lower body weight when mice were fed HFD, but this was not accompanied by a decrease in food intake or enhanced energy expenditure. Neither did developmental NtsR1 deletion alter operant responding for sucrose (a DA-dependent behavior), or anxiety as assessed by multiple measures. This lack of obvious metabolic phenotype after developmental deletion of a neuropeptide receptor is not uncommon (Palmiter et al., 1998), and compensatory changes occurring in early development may offset the impact of lacking NtsR1. However, developmental deletion of NtsR1 from DA neurons in chow-fed mice did increase basal locomotor activity and prolonged AMPH-induced activity, similar to whole body NtsR1 knockout mice with a hyperactive DA system (Liang et al., 2010). These results suggest that deleting NtsR1 from DA neurons during early life may potentiate DA signaling, akin to compensatory increases in extracellular DA that occur with partial loss of DA neurons (Robinson et al., 1994). Numerous mechanisms could enhance DA action, such as altered balance of D1/D2 protein expression in the striatum or impaired DAT kinetics and will be important to define in the future. Curiously, however, when exposed to obesogenic diet, mice with developmental deletion of NtsR1 from DA neurons exhibit no alterations in locomotor activity. It is possible that enhanced DA system hyperactivity in developmental deleted NtsR1 mice was offset by obesity, which can blunt the DA system (Fulton et al., 2006; Sharma and Fulton, 2013; Hryhorczuk et al., 2016). Notably, mice with developmental deletion of NtsR1 from DA neurons were somewhat protected from diet-induced weight gain, though this was not explained by reduced food intake or elevated physical activity. There is evidence that VTA DA neurons may suppress sympathetic regulation of brown adipose tissue and thermogenesis (Brizuela et al., 2019). Going forward, it will be important to examine whether NtsR1-expressing DA neurons can engage the sympathetic nervous system in this way to impact body weight independent of ingestive and locomotor behavior. Our finding that developmental deletion of NtsR1 from DA neurons partially protects mice from HFD-induced weight gain differs from our previous report that whole-body, male NtsR1 knockout mice are prone to overconsuming palatable food and weight gain (Opland et al., 2013). It is possible that NtsR1 expression outside of DA neurons is responsible for overconsumption, and it will be important to assess the role of NtsR1 in other cell populations. It is also important to note that crossing *NtsR1^flox/flox^* and *DAT^Cre^* mice leads to Cre-mediated developmental deletion of NtsR1 from all DA neurons, including those of the VTA and substantia nigra, which may explain the bias of attentional/arousal effects in this line. Although we did not observe different effects in males and females (data is pooled from both sexes), DA signaling can differ in males and females (Zachry et al., 2021), and it is possible that hormonal or environmental context could influence the role of NtsR1-DA neurons, which should also be explored in the future.

Nts treatment in the VTA and chemogenetic activation of adult VTA NtsR1 promotes weight loss (Perez-Bonilla et al., 2021), hence, we anticipated that loss of NtsR1 from adult DA neurons would promote the opposite effects. To our surprise, deleting NtsR1 from the adult VTA also promoted lower body weight independent of *ad libitum* food intake. On its face this lean phenotype is like that produced by adult-onset ablation of VTA NtsR1 neurons. However, mice with ablated VTA NtsR1 neurons were lean primarily due to hyper-locomotor activity and metabolism, and in fact overconsumed food in an attempt to counteract their excessive energy expenditure (Woodworth et al., 2017a). By contrast, our current work deleting just the NtsR1 from intact VTA DA neurons results in a more modest protection from age or HFD-induced weight gain without hyperactivity or overconsumption. It stands to reason that deleting a single GPCR would produce a less robust phenotype compared to killing an entire population of neurons. Yet, the modest impairments in body weight and fasting-induced refeeding observed after adult-onset NtsR1 deletion in the VTA suggests that NtsR1 plays a role in tuning appropriate energy intake behavior and body weight.

Since food deprivation increases DA neuron excitability, decreases DA re-uptake, and potentiates DA release during re-feeding (Hernandez and Hoebel, 1988; Wilson et al., 1995; Ahn and Phillips, 1999; Zhen et al., 2006; Sevak et al., 2008; Branch et al., 2013), we tested whether fasting exposed any alterations in DA-mediated refeeding in mice with developmental or adult-mediated NtsR1 deletion from DA neurons. While developmental deletion of NtsR1 had no impact on this behavior, *VTAR1^Null^* mice with adult-onset NtsR1 deletion from DA neurons had restrained re-feeding compared to controls with intact NtsR1. Similarly, mice with ablated VTA NtsR1 neurons also had impaired ability to coordinate reduced energy status with appropriate feeding (Woodworth et al., 2017a). Taken together, these data support the idea that NtsR1 in established VTA neurons primarily impacts DA-mediated, motivated feeding behavior, which would not be at play during *ad libitum* feeding. Modulating NtsR1 expression or signaling may conceivably be useful to tune DA-dependent feeding without disrupting homeostasis, which could make it a safer approach for modifying behavior. However, since modulating VTA DA signaling has been implicated in weight loss by promoting anxiety, aversion or reinforcement (Seibenhener and Wooten, 2015; Scheggi et al., 2018), we also evaluated whether deleting VTA NtsR1 causes such adverse DA-associated physiology. Notably, deleting VTA NtsR1 from diet-induced obese mice did not invoke these conditions. This is also consistent with activation of VTA NtsR1 neurons, which also did not invoke anxiety or aversion behaviors (Perez-Bonilla et al., 2021). Taken together, these data support that modulating NtsR1 expression or neuronal activity *via* VTA DA neurons holds potential to beneficially promote weight loss without undesirable psychomotor impact. However, we note that developmental deletion of NtsR1 from DA neurons did enhance basal and stimulant-induced locomotor activity suggestive of altered psychomotor function. The cause of this is yet to be determined, but it is possible that early loss of NtsR1 from all DA neurons causes organizational changes that ultimately influence the function and responsivity of the DA system.

Our hypothesis was that deleting NtsR1 from DA neurons would potentiate feeding and weight gain, yet our results demonstrated the opposite: deletion supported modest reductions in weight gain. A key remaining question is why deleting NtsR1 from adult VTA neurons promoted weight loss, while experimentally activating such neurons also invoked weight loss. This seeming dichotomy may reflect that both methods, deletion and experimental activation, could result in loss of NtsR1 expression. Neuropeptide-mediated activation of neurons can cause internalization of their cognate GPCRs. It is possible that chemogenetic activation of VTA NtsR1 neurons in fact led to diminished NtsR1 expression, as well as invoking other signaling pathways that promoted DA release, and both effects may have contributed to weight reduction. In the current study, deleting NtsR1 from adult VTA NtsR1 neurons does not enable any NtsR1-mediated activation, and so may reveal only the contributions of losing GPCR signaling *via* NtsR1. Going forward it will be vital to test how agonist binding to NtsR1 impacts signaling in VTA DA neurons, and whether it biases for sustained intracellular signaling or NtsR1 internalization. Answering these questions is important to understand the mechanism by which NtsR1 contributes to energy balance and how its’ signaling might be modified to bias for weight loss. Nonetheless, our findings show that modulating NtsR1 expression in DA neurons disrupts regulation of energy balance and points to further exploration of the underlying mechanisms.

## Data availability statement

The raw data supporting the conclusions of this article will be made available by the authors, without undue reservation.

## Ethics statement

The animal study was reviewed and approved by the Institutional Animal Care and Use Committee (IACUC) at Michigan State University and the Oklahoma Medical Research Foundation, in accordance with the Association for Assessment and Accreditation of Laboratory Animal Care and National Institutes of Health.

## Author contributions

PP-B, MB, and GL contributed to the conception and design of the study. JR-V, PM, and SA carried out germline model experiments. ET-R and MB carried out electrophysical experiments and data analysis. PP-B carried out adult model experiments, performed statistical analysis, and wrote the first draft of the manuscript. ET-R and MB wrote sections of the manuscript. AM and RB generated and genotyped mouse models for all studies. All authors contributed to manuscript revision, read, and approved the submitted version.
