# Supplementary material for: Developmental or adult-onset deletion of neurotensin receptor-1 from dopamine neurons differentially reduces body weight
Source: Front Neurosci. 2022 Sep 23;16:874316. doi: 10.3389/fnins.2022.874316 (PMC9537700; doi:10.3389/fnins.2022.874316)
Supplement: Supplementary file 1 [file Data_Sheet_1.docx]

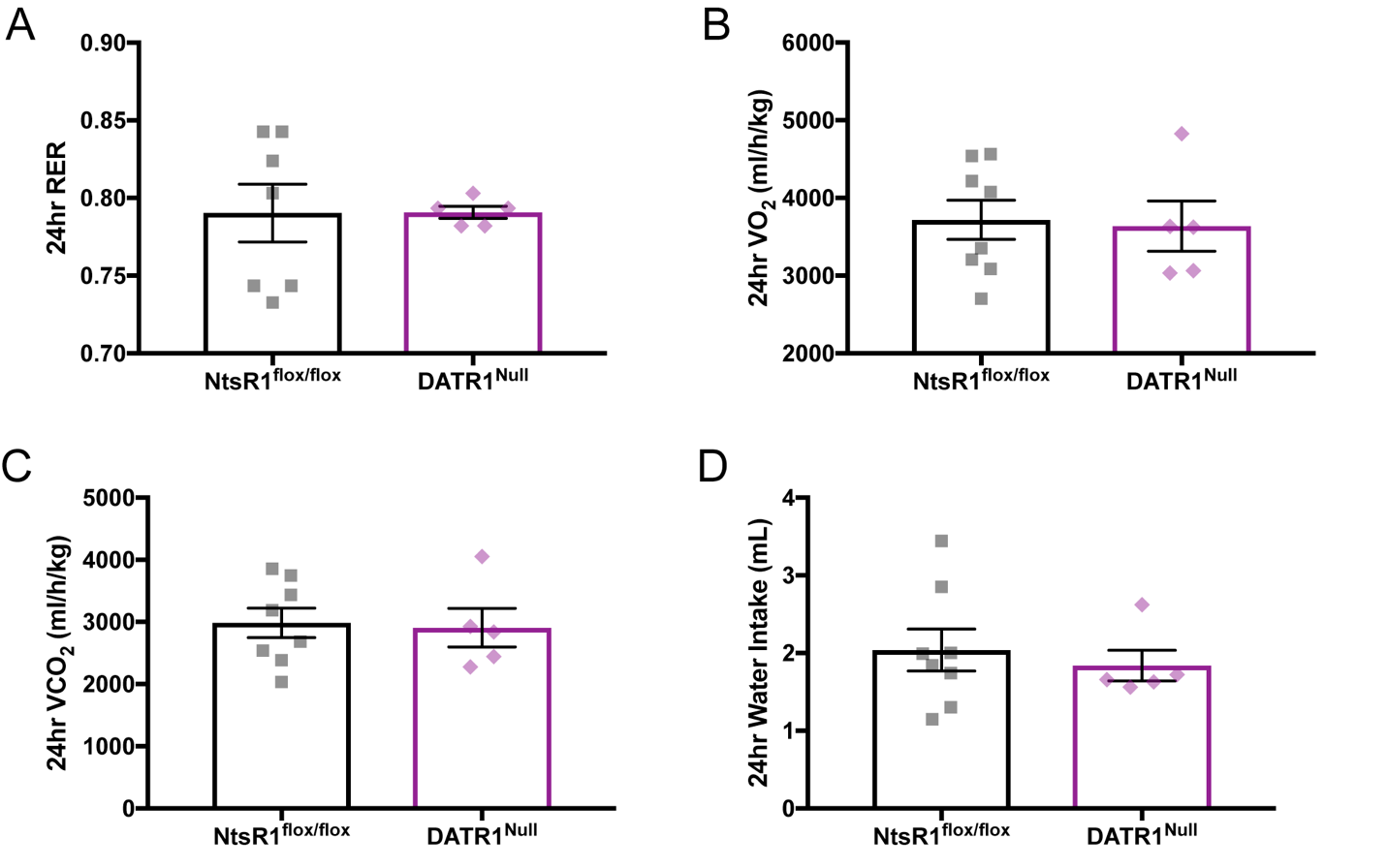


**Supplemental Figure 1. Developmental Deletion of NtsR1 in Diet-Induced Obesity Does Not Alter Energy Expenditure.**  HFD fed *NtsR1^flox/flox^* (n=8) and *DATR1^Null^* (n=5) mice were individually housed and analyzed in metabolic cages. NtsR1^flox/flox^ and DATR1^Null^ mice exhibited comparable energy expenditure over 24 hr, including A) RER, B) VO_2 ,_ and C) VCO_2_ . Mice also had comparable intake of food (not shown) and D) water over 24 hr. No differences were observed in any of these measures across the light or dark cycle (not shown).


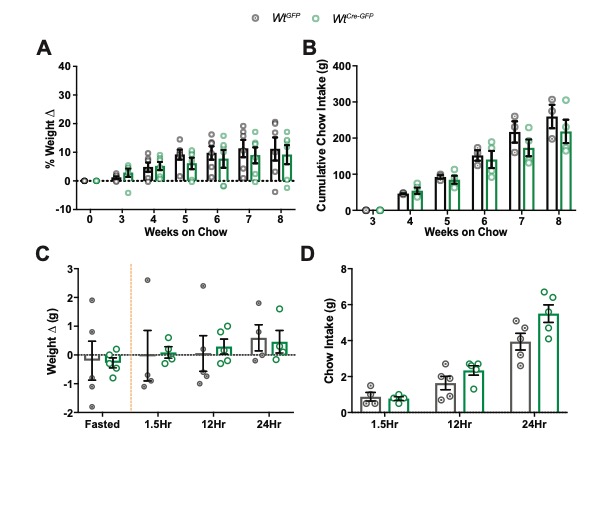


**Supplemental Figure 2. AAV-Cre-GFP does not Alter Body Weight, Chow Intake, or DA-Dependent Chow intake in *Wt* mice.** Injected *Wt* mice were individually housed right after surgery and their chow and body weight was taken every week until the 8-week mark after surgery. A) % Weight change from surgery day to week 8 on chow and B) cumulative chow intake. *Wt* mice injected with AAV-GFP or AAV-Cre-GFP do not significantly differ from each other neither in body weight gain or food intake. Data represent mean ± SEM analyzed via ordinary 2Way-ANOVA with Sidak’s correction (n=4-5). Injected *Wt* mice were fasted overnight and then re-fed the next day with Chow to assess whether AAV-Cre-GFP alters DA-mediated food intake. Weight was measured ~15 hr after mice were fasted and 1.5, 12, and 24 hours after food was restored. C) Weight change before and after normal chow was restored. D) Chow intake after chow was restored. No significant differences between injected groups were observed. Data represent mean ± SEM analyzed via ordinary 2Way-ANOVA with Sidak’s correction for multiple comparisons. n=4-5. *p<0.05.


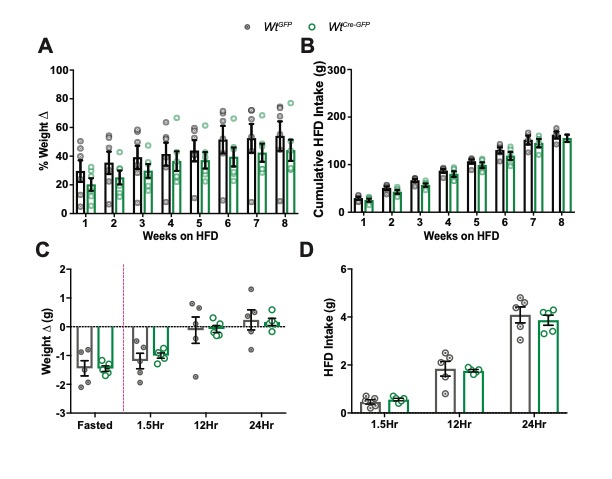


**Supplemental Figure 3. AAV-Cre-GFP does not Alter DA-dependent Food Intake or Weight Gain in *Wt* mice.** Individually housed Injected *Wt* mice were given HFD and their chow and body weight was measured every week until for 8 weeks. A) % Weight change from week 1 to week 8 on HFD and B) cumulative HFD intake. *Wt* mice fed a HFD and injected with AAV-GFP or AAV-Cre-GFP do not significantly differ from each other neither in body weight gain or HFD intake. Data represent mean ± SEM analyzed via ordinary 2Way-ANOVA with Sidak’s correction (n=5). Injected *Wt* mice were then fasted overnight and then re-fed the next day with HFD to assess whether AAV-Cre-GFP alters DA-mediated food intake. Weight was measured ~15 hr after mice were fasted and 1.5, 12, and 24 hours after food was restored. C) Weight change before and after HFD was restored. D) HFD intake after HFD was restored. No significant differences between injected groups were observed. Data represent mean ± SEM analyzed via ordinary 2Way-ANOVA with Sidak’s correction for multiple comparisons. n=5. *p<0.05.


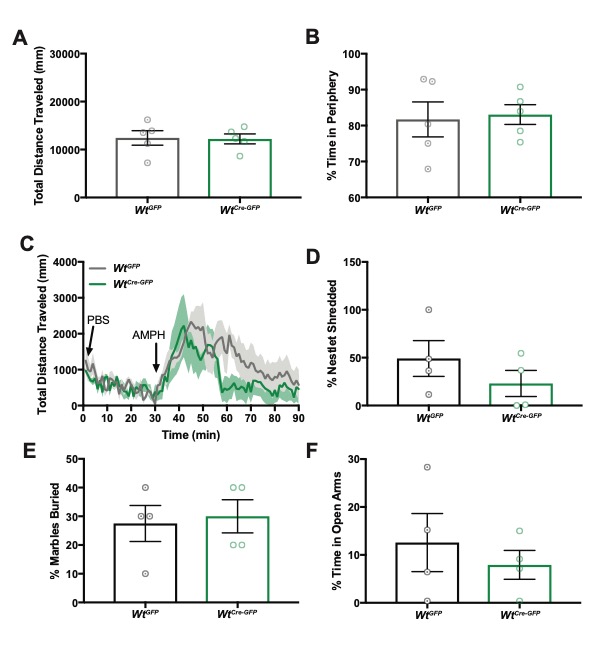


**Supplemental Figure 4.** **AAV-Cre-GFP does not Alter DA-dependent locomotor Activity or Anxiety behaviors.** Injected *Wt* mice were placed in open field boxes and videotracked to assess exploratory behavior and locomotor activity. In A and B, mice were left to explore box for 30mins, bars represent last 15 minutes of data collected and analyzed. In C, mice were left to explore the box for 30 minutes after PBS injection, and for 30 minutes after amphetamine (AMPH) treatment. A) Total distance traveled under baseline condition (no injection). B) % time spent in box periphery under baseline condition. C) Total distance traveled before, during and after PBS and AMPH treatment. AAV-Cre-GFP Injected *Wt* mice did not have a significantly different response to AMPH compared to AAV-GFP Injected *Wt* mice. AMPH data represent mean ± SEM analyzed via ordinary 2-way ANOVA with Sidak post-tests (n=4). To assess whether AAV-Cre-GFP altered anxiety-like behaviors, mice were given a nestlet to shred and marbles to bury for 30 minutes and placed in the Elevated Plus Maze (EPM) for 5 minutes. AAV-Cre-GFP Injected *Wt* mice did not have a significantly different response to D) % of nestlet shredding, E) % of marbles buried, or F) % time spent in open EPM arms when compared to AAV-GFP Injected *Wt* mice. Open field, nestlet shredded, marbles buried and EPM data represent mean ± SEM analyzed via unpaired TTEST (n=4). *p<0.05.
